# Can the success of digital super-resolution networks be transferred to passive all-optical systems?

**DOI:** 10.1515/nanoph-2025-0294

**Published:** 2025-09-08

**Authors:** Matan Kleiner, Lior Michaeli, Tomer Michaeli

**Affiliations:** Faculty of Electrical and Computer Engineering, Technion, Haifa, Israel; School of Electrical & Computer Engineering, Faculty of Engineering , Tel Aviv University, Tel Aviv, Israel; Light Matter Interaction Center, Tel Aviv University, Tel Aviv 6997801, Israel

**Keywords:** diffractive neural networks, all-optical super-resolution, nonlinear optical computing

## Abstract

The deep learning revolution has increased the demand for computational resources, driving interest in efficient alternatives like all-optical diffractive neural networks (AODNNs). These systems operate at the speed of light without consuming external energy, making them an attractive platform for energy-efficient computation. One task that could greatly benefit from an all-optical implementation is spatial super-resolution. This would allow overcoming the fundamental resolution limitation of conventional optical systems, dictated by their numerical aperture. Here, we examine whether the success of digital super-resolution networks can be replicated with AODNNs considering networks with phase-only nonlinearities. We find that while promising, super-resolution AODNNs face two key physical challenges: (i) a tradeoff between reconstruction fidelity and energy preservation along the optical path and (ii) a limited dynamic range of input intensities that can be effectively processed. These findings offer a first step toward understanding and addressing the design constraints of passive, all-optical super-resolution systems.

## 1 Introduction

The spatial resolution of conventional optical systems, such as microscopes, telescopes, and cameras, is fundamentally constrained by their numerical aperture (NA) and the wavelength of light. These systems act as low-pass filters, attenuating high spatial frequencies and thus limiting the ability to resolve fine details [1]. Overcoming this diffraction limit has been a longstanding challenge across numerous scientific fields, from biology [2] to astronomy [3]. Most existing super-resolution methods modify the acquisition process to allow measuring high spatial frequencies and use linear optics to collect light. For example, some super-resolution techniques rely on near-field measurements of the imaged object, extracting high-frequency information encoded in evanescent waves using metametrials [4], [5]. Methods like structured illumination [6], [7], localization microscopy [8], [9], [10], and optical stethoscopy [11] trade temporal resolution for spatial resolution. Other techniques exploit super-oscillatory phenomena [12], [13] or the statistical behavior of speckle and scattering media [14], [15], [16], [17]. However, the requirement to modify the acquisition process poses severe limitations, rendering these techniques impractical for many real-world scenarios.

In contrast to optical techniques, digital super-resolution methods attempt to computationally recover the lost high frequencies. This is done by exploiting prior knowledge on the typical behavior of high-resolution images in the domain of interest (*e.g.*, cellular organelles [18], galaxies [19], natural scenery images [20], etc.). Over the last decade, this field has seen significant advancements thanks to the adoption of deep learning methods [18], [20], [21], [22], [23], [24], [25], [26]. Unlike classical computational methods, such as Richardson–Lucy deconvolution [27], [28] and its extensions, which only enforce simple priors on the restored image (*e.g.*, smoothness), deep learning approaches leverage the ability of neural networks to learn complex nonlinear mappings between low-resolution images and their high-resolution counterparts based on training examples [20]. Specifically, these methods implicitly extract complex image priors from the training data (*e.g.*, the valid shapes of handwritten letters) and thus achieve state-of-the-art reconstruction accuracy, as well as robustness to noise [29]. Unfortunately, however, state-of-the-art digital super-resolution networks are often challenging to deploy on end devices due to their intense computational requirements [30], [31], [32]. Recently, it has been recognized that many computational tasks that can be performed with digital networks can also be effectively implemented all-optically [31], [33], [34], [35], [36]. In particular, all-optical diffractive neural networks [34] emerged as a promising tool for visual information processing. Here, we numerically investigate whether all-optical diffractive networks, using only passive elements, can replicate the success of digital super-resolution networks.

We show that while passive diffractive networks can indeed enhance resolution all-optically, they suffer from two fundamental tradeoffs that are absent in digital networks: (i) a tradeoff between the reconstruction quality and energy preservation and (ii) high sensitivity to the input global intensity. These two tradeoffs stem from how light propagates and interacts with passive nonlinear media. In particular, each nonlinear layer generates high spatial frequencies that correspond to waves propagating at large angles. Some of these may inevitably escape the next layer’s collection cone, thus causing a loss of energy. Additionally, while images in the digital domain are normalized, natural scenes may exhibit a large range of intensities that correspond to markedly different regions of the nonlinearity. This limits the system’s applicability under changing illumination conditions.

## 2 Passive all-optical super-resolution neural network

**Figure 1:**
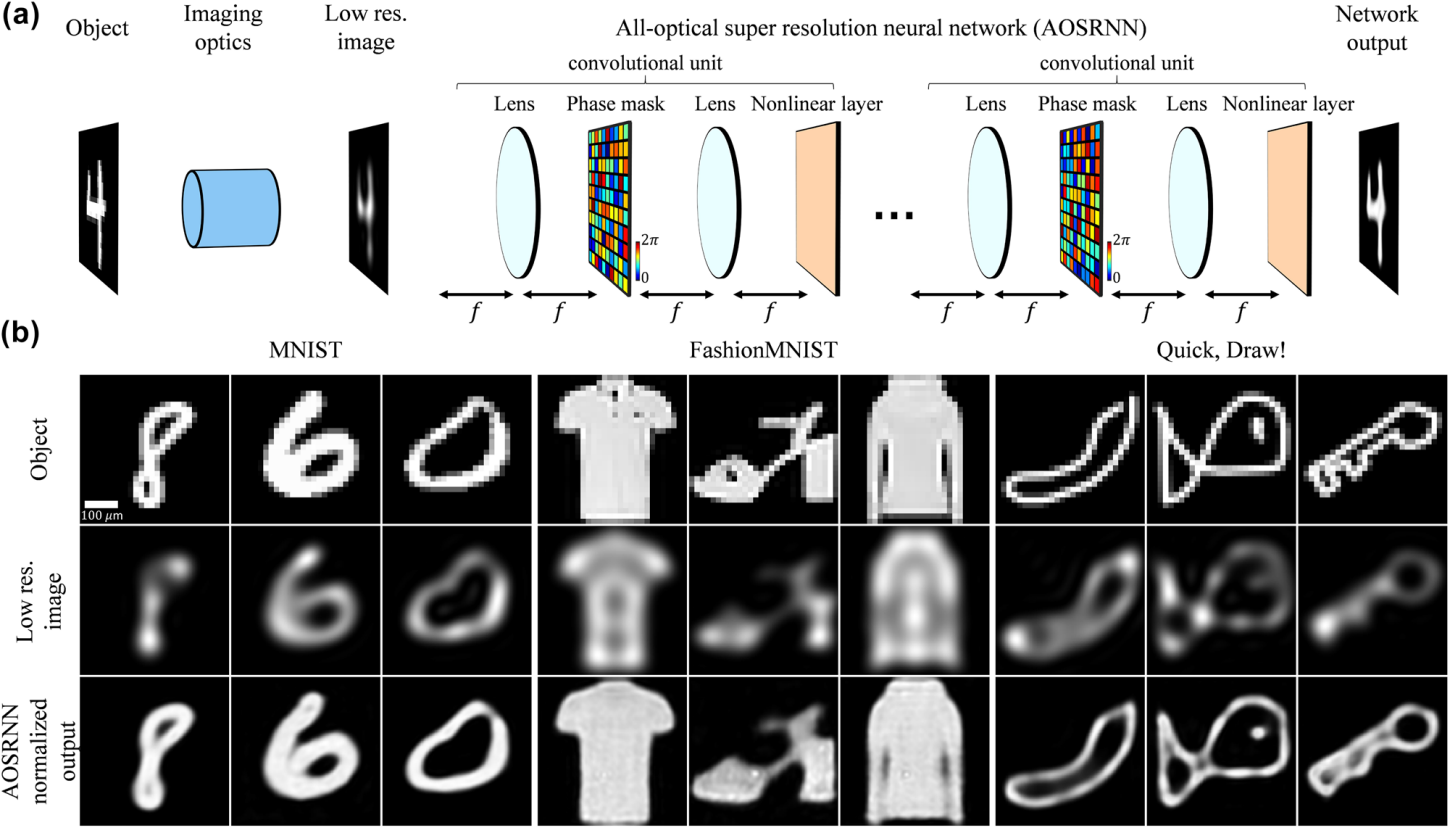
All-optical super-resolution neural architecture (AOSRNN). (a) Schematic of the proposed network. An optical imaging system captures an object and produces a low-resolution image, limited by its NA, which serves as the input to the AOSRNN. This image is then processed by the all-optical neural network, which outputs a high-resolution reconstruction. The network is composed of consecutive convolutional units, each consisting of a 4*f* system with a learnable phase mask layer in the Fourier plane, followed by a nonlinear optical layer. (b) Qualitative results on the MNIST, FashionMNIST, and Quick, Draw! datasets. The first, second, and third rows display the high-resolution object, low-resolution network input, and high-resolution network output, respectively. We note that the ripples observed in the low-resolution images result from the use of a low-NA imaging system with spatially and temporally coherent illumination.

The principles we would like to explore are not inherent to a particular passive diffractive network architecture. However, for concreteness, we focus on an all-optical super-resolution neural network (AOSRNN) architecture inspired by digital convolutional neural networks (CNNs), as illustrated in Figure 1a. This network consists of a series of diffractive convolutional units, each comprising a 4*f* system with a learned phase mask in the Fourier plane and an all-optical nonlinear layer at the image plane. We assume energy-preserving, phase-only nonlinear layers, whose refractive index *n* at spatial location (*x*, *y*) depends nonlinearly on the intensity *I*(*x*, *y*) at that location, as

(1) $$n\left(x,y\right)=n_{0}+n_{2}I\left(x,y\right).$$

Here, *n*_0_ is the linear refractive index, *n*_2_ is the nonlinear refractive index, and *I*(*x*, *y*) = |*E*(*x*, *y*)|^2^ is the intensity of the optical field, which we assume to be monochromatic [37]. While we focus here on phase-only nonlinearities, similar considerations apply to amplitude nonlinearities, which we analyze in Supplementary Note 1. It is important to note that achieving nonlinearity with sufficiently large *n*_2_ is a challenge in its own right and would come at a cost of slow response time. However, the goal of this paper is to illustrate that even if such nonlinear layers were available, other phenomena would still severely limit the network’s utility (see Supplementary Note 1 for further discussions).

The input plane of AOSRNN contains a low-resolution image of an object, obtained from some imaging optics with small NA (left part of Figure 1a). This input image corresponds to a low-pass filtered version of the object, which we assume is illuminated by coherent light. The goal of AOSRNN is to reconstruct the object at its output plane, which coincides with the plane of the last nonlinear layer. Achieving this requires generating spatial frequencies higher than those present at the input image plane, a task facilitated by the nonlinear layers.

To explore the ability of AOSRNN to enhance spatial resolution, we simulated a network composed of 10 convolutional units (see Supplementary Note 2 for the effect of the network’s depth). The input to the network is a low-resolution image, generated by imaging optics with NA of approximately 0.01.

Throughout the paper, we assume that we do not have access to the imaging optics, which produces images of a given resolution. Therefore, our goal is to enhance the input image resolution (relative improvement rather than absolute resolution) by placing the AOSRNN after the imaging system. Assuming a wavelength of *λ* = 550 nm, this corresponds to a cutoff spatial frequency of $f_{c}=\frac{\text{NA}}{\lambda }=\frac{0.01}{550\text{ nm}}=19,345.2\;\;{\text{m}}^{-1}$ , which sets the resolution at the input plane to Δ*x* = 1/*f*_*c*_ = 51.7 μm. We examined a case in which AOSRNN can potentially improve resolution by a factor of up to 4.3. To support this, the apertures of the Fourier plane convolutional units (*i.e.*, the phase mask sizes) were chosen to correspond to a resolution of 12 μm, which translates to a cutoff frequency of *f*_*c*_ = 83,333.3 m^−1^. This represents the highest possible resolution achievable at the network’s output. As shown below, AOSRNN is capable of improving the input image resolution, albeit not fully reaching this theoretical limit. AOSRNN was trained by minimizing the discrepancy between the object intensity, *I*(*x*, *y*), and the intensity at the network’s output, $\hat{I}\left(x,y\right)$ . For simplicity, we represent these quantities as column vectors, omitting their coordinates. We used the *ℓ*_1_ norm between the object and the normalized network output as our loss function. Specifically, let *I* ^(*i*)^ denote the *i*th example in the training set and ${\hat{I}}^{\left(i\right)}$ denote the corresponding network prediction. We constructed a normalized variant of ${\hat{I}}^{\left(i\right)}$ by dividing it by its maximal value and multiplying it by the maximal intensity value across all objects in the dataset, namely ${\hat{I}}_{\mathrm{norm}}^{\left(i\right)}=c\cdot {\hat{I}}^{\left(i\right)}$ , where $c=\frac{\max_{j}\|I^{\left(j\right)}{\|}_{\infty }}{\|{\hat{I}}^{\left(i\right)}{\|}_{\infty }}$ . We then defined the training loss as

(2) $$L=\frac{1}{N}\overset{N}{\underset{i=1}{\sum }}\|{\hat{I}}_{\mathrm{norm}}^{\left(i\right)}-I^{\left(i\right)}{\|}_{1},$$

where *N* is the number of training samples. The normalization was chosen to address the decrease in image intensity that accompanies resolution enhancement, a key challenge that will be thoroughly discussed in the remainder of the paper.

Figure 1b shows several normalized results obtained from three different AOSRNNs, one trained on the MNIST dataset of handwritten digits [38], one on the FashionMNIST dataset of fashion items [39], and one on a subset of the Quick, Draw! dataset, which contains drawings of everyday objects [40]. All three networks effectively recover high-resolution images from their low-resolution counterparts.

**Figure 2:**
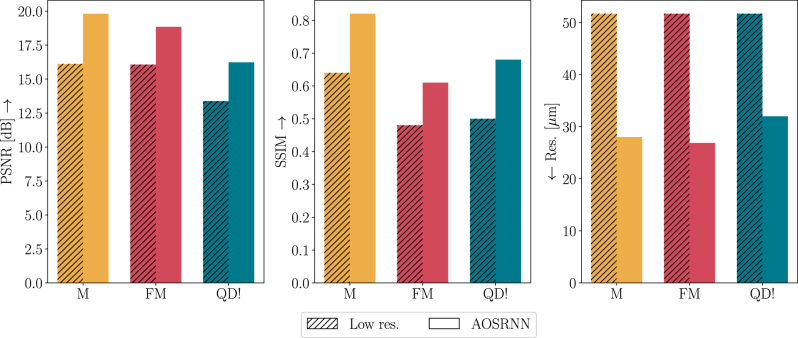
Quantification of resolution enhancement by the all-optical network. Bar plots compare the similarity between the original object and both the low-resolution input (diagonal-striped bars) and the AOSRNN output (solid bars). Similarity is quantified using three metrics: PSNR (left), SSIM (middle), and effective resolution (right). Results are shown for three datasets: MNIST (M), FashionMNIST (FM), and Quick, Draw! (QD).

Figure 2 reports a quantitative evaluation of AOSRNN’s reconstruction quality. The left and middle panes show two measures of discrepancy between the network’s outputs and the original objects: the peak signal-to-noise ratio (PSNR) and the structural similarity index measure (SSIM) [41]. For both metrics, higher scores indicate greater similarity. As can be seen, the similarity between the network’s outputs and the ground-truth images is greater than the similarity between the input low-resolution images and the ground-truth images, suggesting that AOSRNN successfully improves resolution. The right pane of Figure 2 provides a more direct measure of resolution improvement. To quantify resolution, we look for a linear, NA-limited imaging system that achieves the same average PSNR as AOSRNN. We then define the resolution of AOSRNN as the inverse of the cutoff frequency of this linear imaging system (see Supplementary Note 3 for more details). In this case, lower values correspond to better resolution. The bar plot confirms that AOSRNN improves the resolution of the input, with an average enhancement factor of 1.8. However, we note that this improvement is still below the theoretical maximum factor of 4.3, which corresponds to the ultimate resolution bound of 12 μm. We additionally evaluated AOSRNN’s performance using input images generated by imaging optics with NAs of approximately {0.006, 0.004, 0.003}, corresponding to input resolutions of Δ*x* = {84, 134.4, 168} μm. For these cases, AOSRNN enhances resolution by an average factor of {2, 2.6, 3}, respectively. See Supplementary Note 4 for more details.

## 3 Fundamental tradeoffs

As demonstrated above, assuming the availability of suitable nonlinear layers, all-optical diffractive networks, like AOSRNN, can enhance resolution all-optically. However, as we now illustrate, nonlinear optical super-resolution systems suffer from fundamental tradeoffs that are absent in digital networks. In the following subsections, we discuss two such tradeoffs, both inherently tied to the properties of light and thus expected to persist in any type of passive all-optical neural network, regardless of the specific implementation of the nonlinearity. However, we discuss how the working point can be chosen along the tradeoffs.

### 3.1 Resolution versus energy preservation

Our first observation is that, while the nonlinear AOSRNN achieves impressive reconstruction performance, it does so at the cost of significant optical power loss. Only a small fraction of the input field energy is preserved at the output plane of the network, just a few percent (~6%) in the example shown in Figure 1b. This energy loss is not specific to the architecture of AOSRNN but is instead inherent to the physics of light interacting with nonlinear optical layers. Specifically, as light propagates through such systems, the nonlinear layers generate new high spatial frequencies, corresponding to larger diffraction angles. Due to the limited numerical aperture (NA) of the system, some of these high-angle components lie outside the collection cone of the subsequent layers and are thus inevitably lost.

**Figure 3:**
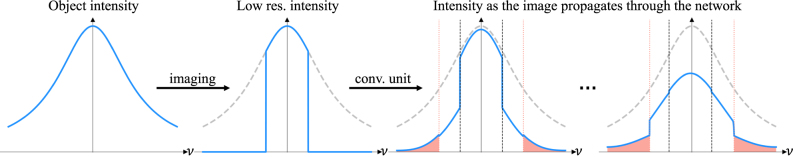
Energy loss in nonlinear networks. Illustration of wave propagation through a nonlinear network in the spatial frequency domain, plotted along one spatial dimension as a function of spatial frequency *ν*. An imaging system with a low NA generates the low-resolution input image, effectively applying a low-pass filter *H*. The black dashed vertical lines mark the NA limit of this original system. As the wave propagates through the network, nonlinear interactions generate higher spatial frequencies, enabling partial reconstruction of previously lost high-frequency information, up to the NA defined by the collection cones of the convolutional units (red vertical dotted lines). Inevitably, this nonlinear spectral broadening also produces components beyond this NA, resulting in energy loss due to spectral content leaking outside the network’s collection cone, highlighted by the red shaded regions. Consequently, the spectral intensity at low frequencies diminishes as the wave progresses through the network. At the final layer, the spectrum within the network’s NA provides a scaled approximation of the original object, represented by the dashed gray line in all plots. The diagrams show the spatial frequency content at the frequency plane of each convolutional unit, just before its corresponding phase mask.

This effect is illustrated in Figure 3, using a simplified one-dimensional example. The spatial frequency content of a typical object naturally decays with increasing spatial frequency (leftmost plot). A low-resolution version of the object is obtained by the imaging optics that precede the network, effectively acting as a low-pass filter that suppresses frequencies beyond the system’s NA (second plot).

As the field propagates through the network, nonlinear interactions give rise to new spatial frequency components that were not present in the initial low-resolution image. These new high-frequency components are crucial for recovering fine spatial details of the original object. The convolutional units act to shape this growing frequency content, steering the evolution of the field toward a high-fidelity reconstruction of the object up to a global normalization constant, as formalized by Eq. (2). However, the newly generated high-frequency components correspond to light propagating at large angles. Due to the finite NA of the network’s convolutional units, some of these components fall outside the collection cone of subsequent layers (red dotted vertical lines) and cannot be captured. This excess bandwidth is inevitably lost, as illustrated by the red shaded regions in Figure 3. As the field continues to propagate through the nonlinear network, each layer builds upon the previous one, reconstructing a progressively higher-resolution approximation of the object but at the cost of cumulative energy loss at every stage. A detailed frequency-domain derivation of this energy leakage is provided in Supplementary Note 5.

**Figure 4:**
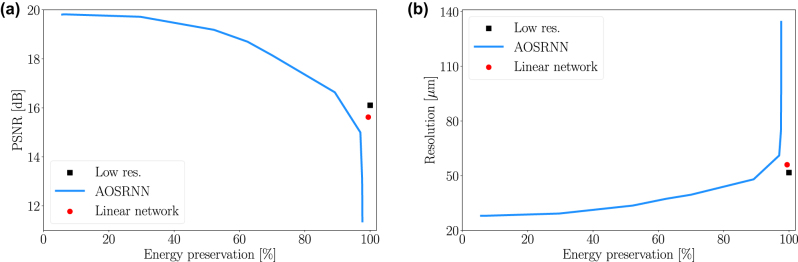
Reconstruction fidelity vs. energy preservation. (a) PSNR in dB and (b) resolution in μm as a function of the fraction of energy preserved at the output (0–100 %). The blue line shows the results of AOSRNN, trained with 12 different values of the regularization parameter *γ* ranging from 10^−1^ to 10^−9^. The black squares indicate the performance of the low-resolution input images. The red dots represent a linear network with the same architecture as AOSRNN but without the nonlinear layers.

This inherent energy loss inevitably leads to output images with low total intensity. Such weak outputs are undesirable, as they are more susceptible to being overwhelmed by noise during the digital acquisition process. A simple approach to improve energy preservation is to amend the loss function of Eq. (2) with a regularization term that penalizes for low output intensities,

(3) $$L=\frac{1}{N}\overset{N}{\underset{i=1}{\sum }}\left(\|{\hat{I}}_{\mathrm{norm}}^{\left(i\right)}-I^{\left(i\right)}{\|}_{1}-\gamma \|{\hat{I}}^{\left(i\right)}{\|}_{1}\right),$$

where the regularization parameter *γ* controls the strength of the energy-preserving term. However, this solution turns out to introduce a strong tradeoff between energy preservation and performance, measured in terms of PSNR and resolution, as illustrated in Figure 4 for the MNSIT dataset. When the regularization parameter *γ* is very small, the network prioritizes image fidelity and achieves its best performance but preserves very little energy. As *γ* increases, the network retains more energy, but this comes at the cost of reduced reconstruction quality. For sufficiently large values of *γ*, the network’s performance degrades to the point where it underperforms even the low-resolution input images, as indicated by the black squares in the figure.

A network that simply transmits low-resolution images to its output plane should, in principle, achieve performance metrics identical to those of the low-resolution inputs. Such a network would effectively reproduce the input field at the output, without modification. However, this is not feasible with a nonlinear architecture, as the nonlinear layers inherently perturb the input during propagation. By removing these nonlinear elements from AOSRNN, the resulting linear network could, in theory, learn to perform this identity mapping. In practice, however, the optimization process rarely converges to this solution. Consequently, even the linear network introduces slight distortions to the input, as indicated by the red dots in Figure 4a and b.

The above approach offers a simple way to improve the preservation of input energy at the cost of reducing reconstruction accuracy for a given network configuration. The tradeoff between energy preservation and reconstruction accuracy can be slightly improved by increasing the network’s NA. For example, doubling the width and height of each layer in a network trained on the MNIST dataset without the energy preservation loss term improves energy preservation from ∼6 % to ∼10 % and reconstruction quality from 19.8 dB to 20.3 dB. However, increasing the physical size of the layers results in a larger system, which may be impractical in some applications. It may also introduce fabrication challenges that limit performance.

An alternative way to increase the NA is by reducing the focal length between the optical elements. This could help retain more input energy while also reducing the system’s physical footprint. In this context, optical metasurfaces offer a promising future direction. Their ability to precisely control local phase, amplitude, and polarization at subwavelength scales enables compact optical components with high NA [42]. As such, metasurfaces are an appealing platform for compact, energy-efficient optical neural networks and analog computing systems [43], [44], [45]. Notably, our modeling framework is general and directly applicable to metasurface-based implementations, since all optical components in the network are defined by their local phase profiles, regardless of physical realization.

### 3.2 Sensitivity to global intensity

Our second key observation relates to the sensitivity of the network to the global input intensity. In the digital domain, images are typically normalized to the range [0, 1]. However, in natural scenes, illumination intensity can vary across different scenarios. While this variation is generally not problematic for linear systems, where output intensities scale proportionally with the input, nonlinear systems behave quite differently. Even slight changes in input intensity can result in dramatically different outputs, as they shift the input field into different regimes of the system’s nonlinearity.

**Figure 5:**
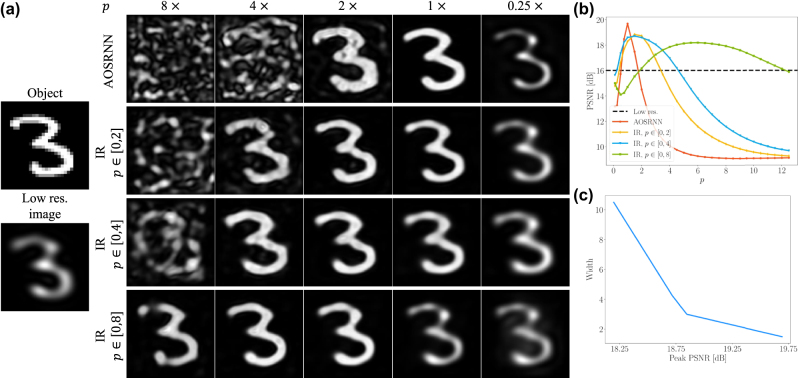
Sensitivity to global intensity. (a) Representative output images from different networks evaluated at different *p* values. The standard AOSRNN fails to generalize to unseen intensity levels, whereas the IR networks produce high-quality reconstructions across varying *p*. (b) Reconstruction fidelity, quantified by PSNR in dB, as a function of the maximal image intensity *p* = max_*j*_|*I* ^(*j*)^|_∞_. The orange curve shows the performance of a standard AOSRNN, while the yellow, blue, and green curves show the results of an intensity-robust (IR) version of AOSRNN, each one trained with *p* values in different range. The black dashed line indicates the PSNR of the low-resolution input images. While the IR networks maintain consistent performance across a wide range of *p* values (denote as width), the regular network shows a sharp decline in performance when *p* ≠ 1. (c) The blue curve denotes the inherent tradeoff between reconstruction fidelity (horizontal axis, measured by peak PSNR) and stability to different peak intensity values (vertical axis, measured by width).

Figure 5 illustrates the results of a network trained on the MNIST dataset with maximal intensity value, *p* = max_*j*_‖*I* ^(*j*)^‖_∞_, of 1, when evaluated on inputs with varying maximal intensities, ranging from 0.1 to 12.5. The results show significant degradation, both quantitatively and qualitatively, when the input intensity deviates from the training value. For example, increasing the intensity by a factor of 2 leads to a drop of approximately 5 dB in PSNR, as shown by the orange curve in Figure 5b. Qualitative deterioration is also evident in the first row of Figure 5a, where the network fails to generalize to inputs with *p* ≠ 1.

A straightforward approach to accommodating a larger range of intensities is to expose the network to varying *p* values during training [46]. We trained three different networks, where the varying *p* values were in the range [0, 2], [0, 4], [0, 8], indicated by the yellow, blue, and green curves in Figure 5b, respectively. Each of these intensity-robust (IR) networks demonstrate improved stability for different *p* values. Qualitative results for each of these networks are shown in Figure 5a. The IR networks improved stability can be seen by the increased range of input intensities over which their reconstruction quality exceeds the input quality, *i.e.*, the range of *p* values over which the different curves in Figure 5b exceed the black dashed line. We term this range width. However, a larger width comes at the expense of reconstruction fidelity, causing a drop in the peak PSNR achieved by each network. It can be seen in Figure 5b that the peak of the orange curve, representing a network trained with a single *p* value, is the highest. Figure 5c illustrates the inherent tradeoff between reconstruction fidelity (horizontal axis, measured by peak PSNR) and stability to different peak intensity values (vertical axis, measured by width), for passive all-optical nonlinear networks.

## 4 Discussion

We have demonstrated that passive all-optical neural networks are theoretically capable of achieving spatial super-resolution but suffer from two fundamental limitations: an inherent sensitivity to the input intensity and a tradeoff between energy preservation and performance. Although these sensitivities can be reduced by exposing the network to diverse conditions during training, this comes at the expense of deterioration in performance.

As mentioned in Section 2, our analysis, although independent from a specific choice of nonlinear layer, still assumes the availability of such a layer. Achieving an all-optical nonlinear response with sufficiently large *n*_2_ is a challenge in its own right [31]. In Supplementary Note 1, we list various physical mechanisms that could be used to implement such nonlinear layers and discuss their shortcomings. Additionally, we demonstrate that AOSRNN can also be trained with a different nonlinear layer, a hybrid optopelectronic nonlinear device recently introduced by Zhang et al. [47].

**Figure 6:**
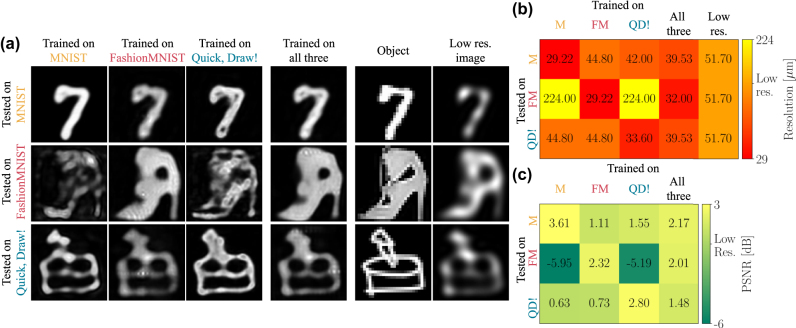
AOSRNN results on out-of-distribution data. (a) Qualitative results of networks trained using the MNIST, FashionMNIST, and Quick, Draw! datasets, as well as a network trained using all these three datasets. These networks were then evaluated on the MNIST, FashionMNIST, and Quick, Draw! datasets. All the results are given next to the objects and the low-resolution images. (b) Resolution results of the different networks when evaluated on the different datasets. (c) PSNR results of all networks w.r.t the low resolution images, when evaluated on the different datasets.

It is instructive to note that, unlike conventional linear optical systems, diffractive networks are also fundamentally restricted by their training data, as they adapt to its underlying statistics. As a result, a network trained on one dataset may generalize poorly to images from another dataset. Figure 6 illustrates this effect using the MNIST, FashionMNIST, and Quick, Draw! datasets (see Supplementary Note 6 for more datasets). As shown, performance on out-of-distribution data highly depends on the degree of distribution shift. For example, networks trained on MNIST or Quick, Draw! datasets fail to generalize to the FashionMNIST dataset, which is characterized by larger smooth regions. In these cases, the results are even worse than the input low-resolution images. Figure 6 further shows the performance of a network trained on all three datasets. When tested on a specific dataset, this network achieves improved results with respect to the networks that were not trained on that dataset but slightly worse results than the network trained on that particular dataset. This highlights yet another fundamental tradeoff in all-optical super-resolution – the more diverse the data on which the network is trained, the smaller the resolution improvement. Supplementary Note 6 provides additional details. Supplementary Note 7 illustrates the cost of achieving immunity to all the discussed effects simultaneously – sensitivity to global intensity, to energy preservation, and to out-of-distribution data.

While this work primarily addresses all-optical spatial super-resolution, the tradeoffs we identify are inherently linked to the properties of light and may thus arise in different tasks as well. For example, when training our AOSRNN on image classification task, it achieves 94.8 % test classification accuracy on the MNIST dataset. However, this performance is achieved by scattering a significant portion of the incoming light. Additionally, this network is sensitive to the global input intensity, experiencing a 33 % drop in classification accuracy when increasing the input intensity by a factor of 2 (see Supplementary Note 8 for further details).

The fundamental limitations of energy-preservation versus resolution and sensitivity to global intensity are also not unique to a specific network architecture or to the choice of nonlinear layers but are instead intrinsic to passive nonlinear optical networks. This is illustrated in Supplementary Note 9, where a nonlinear passive network with a different architecture [34] is trained to perform super-resolution. This network, which comprises of a sequence of diffractive layers interleaved by nonlinear layers (without 4*f* systems), successfully enhances the resolution of the input images, achieving a PSNR of 17.4 dB for input images with a PSNR of 16.1 dB. However, it does so by preserving only ∼1 % of the energy in its output plane. Furthermore, when multiplying the input intensity by 2 or by 4, the achieved PSNR deteriorates to 16.6 dB and 14.2 dB, respectively (see Supplementary Note 9 for further details).

Finally, the fundamental limitations of energy-preservation versus resolution and sensitivity to global intensity are not unique to monochromatic light. This is demonstrated in Supplementary Note 10, where we trained an AOSRNN for objects illuminated by polychromatic light comprising three different wavelengths – {400, 550, 700} nm. This network, again, successfully enhances the input image resolution, yet, still suffers from the identified tradeoff (see Supplementary Note 10 for further details.)

These analyses highlight the need for future designs to carefully consider these tradeoffs and identify operating points that are well suited to the task and deployment context. By exposing and quantifying these limitations, our work marks an important step toward enabling more robust development of passive all-optical neural systems and lays out the key considerations for implementing all-optical super-resolution neural networks.

## 5 Methods

For training AOSRNN, we used a dataset D of objects (images), $D={\left\{I^{\left(i\right)}\right\}}_{i=1}^{N}$ . The input to the network was the low-resolution images of *I* ^(*i*)^, resulting from the imaging optics. We implemented the imaging optics as ideal filtering in the frequency domain [1].

We used the MNIST [38], FashionMNIST [39], and Quick, Draw! [40] datasets, which contain 28 × 28 grayscale images of handwritten digits, fashion items, and drawing of different everyday objects, respectively. In all of the numerical experiments, we first upsampled these images using nearest-neighbor interpolation to 112 × 112 graysacle images. The input images were padded accordingly to prevent aliasing. The MNIST and FashionMNIST datasets include 10 different classes, while the Quick, Draw! dataset includes draws of 345 different classes. For the numerical experiments, we used 10 out of these 345 classes. From the Quick, Draw! dataset, we used the classes of aircraft carrier, banana, cake, diamond, fish, guitar, hexagon, key, microphone, and saxophone.

We trained the different networks with 10,000 training images ( ∼1,000 per class) for 1,000 epochs, using the Adam optimizer [48] with a learning rate of *η* = 1 × 10^−1^. We used a scheduler that reduces the learning rate by a factor of 2 every 100 epochs. The simulations were implemented using the Pytorch deep learning framework [49] and were executed on a Linux machine with NVIDIA GeForce GTX 2080 Ti GPU. Standard training took ∼7 h on this machine. The intensity robust (IR) networks were trained using the same hyperparamters and training process but with maximal intensity values chosen uniformly from the predefined set [0.1, 0.25, 0.5, 0.75, 1, 2, 3, …]. The largest value of this set was either 2, 4, or 8, for the different networks.

For details regarding the forward propagation through AOSRNN, see Supplementary Note 11.

## Supplementary Material

Supplementary Material Details
