# Supplementary material for: Can the success of digital super-resolution networks be transferred to passive all-optical systems?
Source: Nanophotonics. 2025 Sep 8;14(19):3181–90. doi: 10.1515/nanoph-2025-0294 (PMC12455410; doi:10.1515/nanoph-2025-0294)
Supplement: Supplementary file 1 — Supplementary Material Details [file j_nanoph-2025-0294_suppl_001.pdf]

Can the Success of Digital Super-Resolution Networks Be Transferred to Passive All-Optical Systems? – Supplementary Material

Supplementary Note 1 All-optical nonlinear layers

The numerical experiments described in the main text depend on the availability of a nonlinear layer with sufficient nonlinearity. Devising a suitable nonlinear layer for free-space all-optical computation remains an open challenge and an active area of research [1, 2], which is beyond the scope of this work. In this note, we first describe how the nonlinear layers in our AOSRNN implementation are simulated. We then discuss various physical mechanisms that could drive such layers in practice. Finally, we demonstrate that AOSRNN can also be trained with a different nonlinear layer, a hybrid optopelectronic nonlinear device recently introduced by Zhang et al. [3].

1.1 Simulated nonlinear layer in AOSRNN

In our implementation, we focus on energy-preserving and phase-only nonlinearities, whose refractive index n at spatial location (x, y) depends nonlinearly on the intensity $I(x, y)$ at that location, as

$$n(x, y) = n_0 + n_2 I(x, y). \tag{S1}$$

Here, n_0 is the linear refractive index, n_2 is the nonlinear refractive index and $I(x, y) = |E(x, y)|^2$ is the intensity of the optical field, which we assume to be monochromatic [4]. Wave propagation through such a thin nonlinear layer is formulated as

$$E^+(x', y') = E^-(x, y) \cdot e^{-j\frac{2\pi}{\lambda}d(n_0+n_2 I(x,y))}, \tag{S2}$$

where $E^+(x, y)$ and $E^-(x, y)$ are the fields immediately after and before the nonlinear layer, respectively, λ is the wavelength and d is the thickness of the nonlinear layer.

In the simulations, the input intensities were normalized to the range $[0, 1]$. Denoting the intensity in the simulation by I_s (as opposed to the physical intensity I), the phase due to the nonlinear index was taken to be αI_s for some constant $\alpha \in \{0.25, 1, 4\}$. This allows us to retrospectively interpret each simulation result in terms of different pairs of I and n_2. Specifically, the product αI_s corresponds to $\frac{2\pi}{\lambda} d n_2 I$, with $\lambda = 550$ nm and $d = 6$ μm. Thus, we can regard the simulations as corresponding to a real intensity of $I = \beta I_s$ for some factor β with a nonlinear index of $\frac{\alpha\lambda}{2\pi d\beta}$. In other words, the same simulation can be interpreted as corresponding to different real intensities, given that we scale the nonlinear index accordingly. Unless otherwise specified, results reported in this work correspond to $\alpha = 4$.

Figure S1 illustrates the relation between different n_2 values and intensity, for different α values. For instance, assuming a power of $P = 100\,\text{mW}$ and an image size of 0.448 mm $\times$0.448 mm, the results in Fig. 1 in the main text, were achieved using $n_2 \approx 1 \times 10^{-3}$ cm^{2}W^{-1}. While this is a relatively high value, it is attainable through mechanisms such as thermal effects and director reorientation in liquid crystals (LC) (see Tab. 1). These mechanisms typically involve slow response times (on the order of milliseconds) [5]. However, recent progress in material formulations and device architectures has led to substantial improvements in speed. For example, nanocavity-enhanced liquid crystal modulators have demonstrated switching rates nearly two orders of magnitude faster than conventional configurations by confining the LC within nanoscale silicon gratings and maximizing the overlap with the optical field [6]. Moreover, research into platforms utilizing inherently faster nonlinearities—such as epsilon-near-zero (ENZ) materials like indium tin oxide (ITO) [7, 8], has enabled high nonlinear refractive indices with femtosecond-scale response times. Engineered metasurfaces based on hybrid dielectric–plasmonic architectures and high-index Mie-resonant structures have also demonstrated large nonlinearities while maintaining ultrafast temporal dynamics [9, 10] has enabled substantial increases in achievable nonlinearities even for fast, electronic Kerr-based mechanisms. These advances are progressively narrowing the long-standing tradeoff between response speed and nonlinear strength and are likely to play a central role in the realization of practical all-optical nonlinear neural networks.

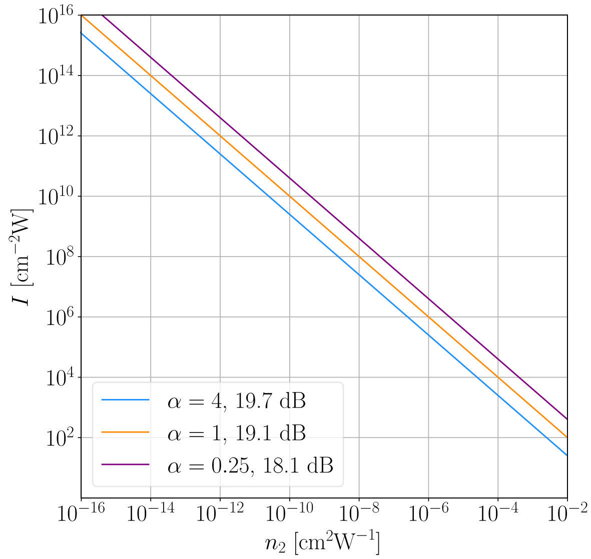

Fig. S1: Reinterpreting simulation results via the constant $n_2 I$ product. Each curve corresponds to a network trained with a different value of the scaling factor α, which determines the product $n_2 I$ and thus the strength of the nonlinearity. The horizontal axis corresponds to n_2 values in cm^{2}W^{-1}, and the vertical axis is the optical intensity in W cm^{-2}. Because performance depends only on the product $n_2 I$, the results of a given simulation (fixed α) can be reinterpreted for any physically meaningful pair of n_2 and I that falls along the corresponding curve. The legend reports the PSNR achieved by each network.

1.2 n_2 of various materials resulting from different nonlinear mechanisms

The n_2 value of many common materials (*e.g.*, fused silica, calcite) due to the optical Kerr effect is $\sim 10^{-16}$ cm^{2}W^{-1} [11, 12]. Using these materials would necessitate beams of very high optical intensity. Other materials have much larger n_2 values, *e.g.*, liquid crystals have n_2 values of $10^{-4} - 10^{-6}$ cm^{2}W^{-1} due to the thermal effect and n_2 values of $\sim 10^{-3}$ cm^{2}W^{-1} due to axis reorientation, but they have their own drawbacks. For example, the response time, which is almost instantaneous for the optical Kerr effect, can be in the order of nanoseconds to microseconds for the thermal effect, and microseconds and more for axis reorientation [4]. Generally, materials with small n_2 values tend to exhibit faster response times, while those with large n_2 values often exhibit slower dynamics [4].

Table 1 summarizes the n_2 values, resulting from different nonlinear mechanisms, for polymers, glass, semiconductors and liquid crystals. The material properties are based on the review paper by Christodoulides et al. [4]. Although stronger nonlinearities have been explored since the publication of this review, the table highlights the order of magnitude of n_2 that can be achieved with different mechanisms. The Polymers category includes conjugated polymers and the Glass category includes fused silica, glasses with metal oxides and chalcogenide glass. Liquid crystals include doped liquid crystals.

Generally, when choosing a nonlinear layer for diffractive networks various aspects need to be taken into consideration. These include locality, efficiency (which in some cases deteriorates with stronger nonlinear effect [4]), working spectrum and task requirements.

1.3 AOSRNN with optoelectronic nonlinear layer

In the main text, we focused on energy-preserving and phase-only nonlinear layers with a nonlinear refractive index. However, other optical nonlinearities, both all-optical and optoelectronic [2], can potentially be used as part of an optical neural network. For example, recent nonlinear optoelectronic devices [3, 13], achieve nonlinearity under low optical power, and are thus appealing candidates. These

Tab. 1: Examples of nonlinear mechanism in different materials and their n_2 values.

Material	Nonlinear mechanism	n_2 [cm^2W^{-1}]
Polymers	Kerr effect	$\sim 10^{-12}$
	Electrostrictive effect	$\sim 10^{-15}$
Glass	Kerr effect	$10^{-13} - 10^{-16}$
	Electrostrictive effect	$\sim 10^{-16}$
Semiconductors	Two-photon absorption	$10^{-7} - 10^{-9}$
	Multiple quantum well	$10^{-6} - 10^{-13}$
	Quantum dots	$10^{-11} - 10^{-14}$
	Kerr effect	$\sim 10^{-13}$
	Electrostrictive effect	$\sim 10^{-15}$
	Thermo-optic Effect	$10^{-10} - 10^{-16}$
Liquid Crystal	Thermal effect	$10^{-4} - 10^{-6}$
	Director reorientation	$10^{-3} - 10^{3}$

nonlinear devices can modulate white LEDs, and therefore could even be used in an all-optical neural network that works under partially coherent light [14], enabling optical spatial super-resolution under ambient light.

As a concrete example, the optoelectronic nonlinear device introduced by Zhang et al. [3] is based on transparent phototransistors with liquid crystal modulators. This device exhibits a nonlinear transmission curve and can be used as the nonlinear layer in an optical nonlinear neural network. It is not energy-preserving, as its nonlinear effect is based on absorption, but can work under relatively low illumination intensities. We simulated this device and its nonlinear transmission behavior, based on the measurements reported by Zhang et al. [3]. Specifically, we used the measurements corresponding to a voltage supply of 6 V (extracted from the brown curve in Fig. 3a of their paper). We fitted a decaying exponent to these measurements, resulting in a function that maps intensity to transmission ratio, $\mathrm{TR}(I)$.

Propagation through a nonlinear layer based on this optoelectronic device is equivalent to

$$E^{+}(x', y') = E^{-}(x, y) \cdot \mathrm{TR}(I(x, y) \cdot t), \tag{S3}$$

where E^{+} and E^{-} are the field just after and before the nonlinear layer, respectively, and t is a parameter that translates the intensity from the normalized values used in the simulation (in the range $[0, 1]$) to units of $\mathrm{mWcm^{-2}}$. We used $t = 200\ \mathrm{mWcm^{-2}}$.

We trained AOSRNN on the MNIST dataset using the same architecture described in the main text, but with this optoelectronic nonlinear device as the nonlinear layers. This network was also trained with the energy preservation regularization term. This network achieves a PSNR of 17.7 dB and resolution of 42 μm with $\sim$15% energy preservation. In comparison, a network trained using the nonlinear layers with the nonlinear refractive index achieves a PSNR of 19.7 dB and a resolution of 29.2 μm, with $\sim$30% energy preservation.

Figure S2a shows a qualitative comparison between these two networks on the MNIST dataset. Figure S2b depicts the resulting phase masks for the two networks.

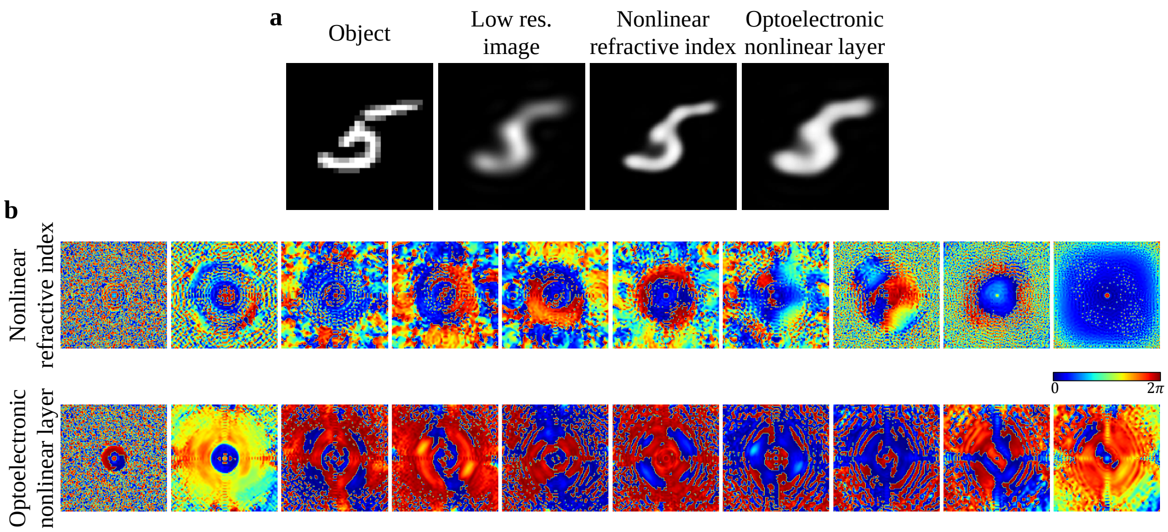

Fig. S2: Comparison between a trained AOSRNN comprising refractive index nonlinear layers and one comprising optoelectronic nonlinear layers. (a) Qualitative super-resolution results on the MNIST dataset. (b) Learned phase masks for the two AOSRNN implementations, with values ranging from 0 to 2π.

Supplementary Note 2 Effect of network depth on the performance of AOSRNN

Figure S3 shows the performance of trained networks of varying depths. The results of a shallow network, consisting of only a single convolutional unit (a $4f$ system with a learned phase mask followed by an all-optical nonlinear layer), do not improve upon the low-resolution input images. As expected, increasing the number of consecutive convolutional units steadily improves both the qualitative and quantitative performances, as can be seen in Fig. S3a,b. Figure S3c displays the learned phase masks of networks with different numbers of convolutional units.

All the numerical experiments discussed in this note were performed with the MNIST dataset and the hyperparameters described in the main text and with the energy preservation regularization term. The images at the input of the networks correspond to imaging optics with NA of approximately 0.01. All networks discussed in this note preserve at least 30% of the input energy.

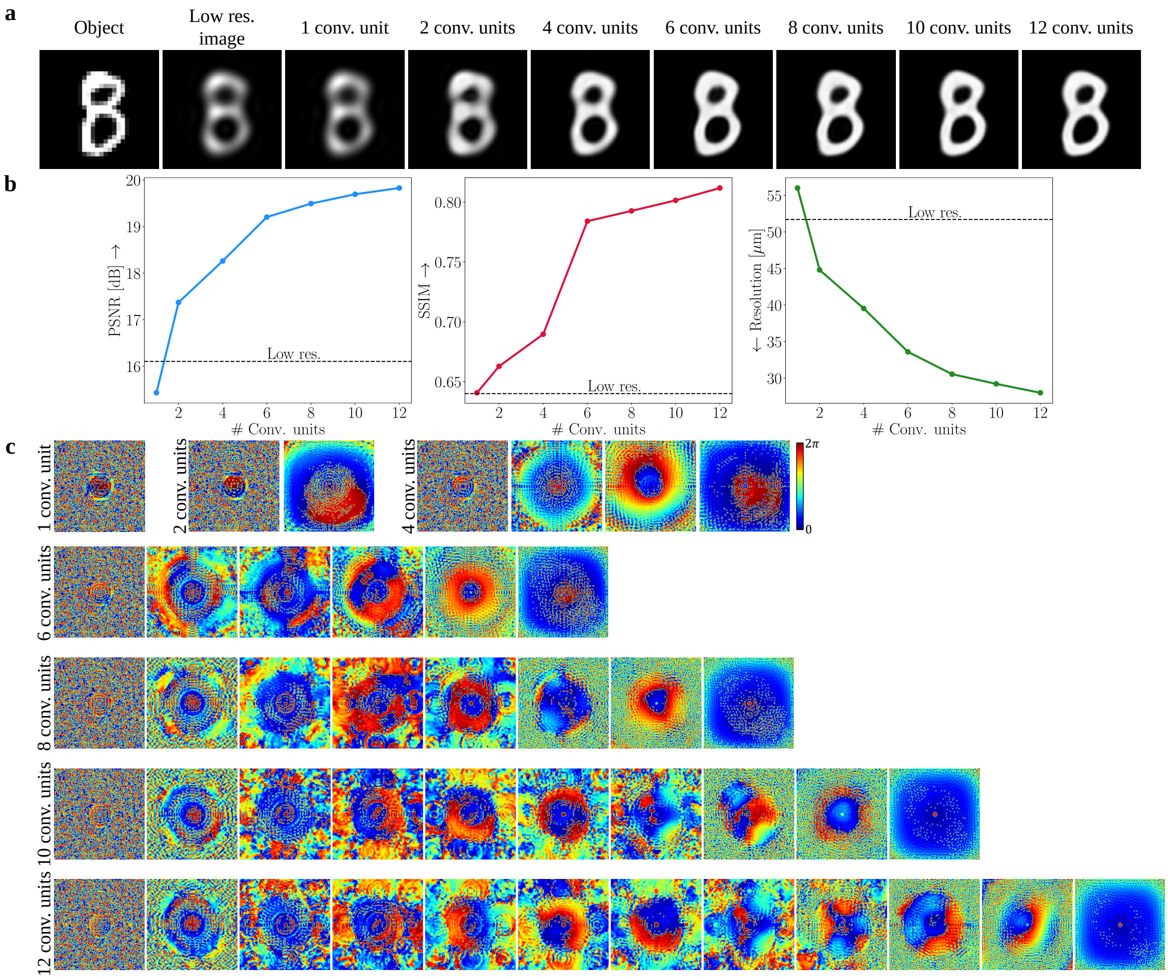

Fig. S3: Effect of depth on AOSRNN performance. (a) Qualitative results of networks with different number of convolutional units. (b) Quantitative results, using PSNR (measured in dB), SSIM and resolution (measured in μm). The black dashed line denote the value of the low resolution images. (c) Learned phase masks of networks with different number of convolutional units, with values ranging from 0 to 2π.

Supplementary Note 3 Resolution measurement for nonlinear optical systems

As mentioned in Sec. 2 of the main text, resolution is measured as the inverse of the cutoff frequency of a linear, NA-limited, imaging system achieving the same average PSNR as the examined AOSRNN. We refer to this imaging system as an equivalent imaging optics. It should be noted that the low-resolution images produced by this equivalent imaging optics may not necessarily be visually similar to the outputs of AOSRNN. Indeed, AOSRNN is a nonlinear system and its outputs cannot generally be accurately mimicked by a linear system. Figure S4 shows a qualitative comparison between the outputs of trained networks and those of equivalent imaging optics, where the images have approximately the same average PSNR.

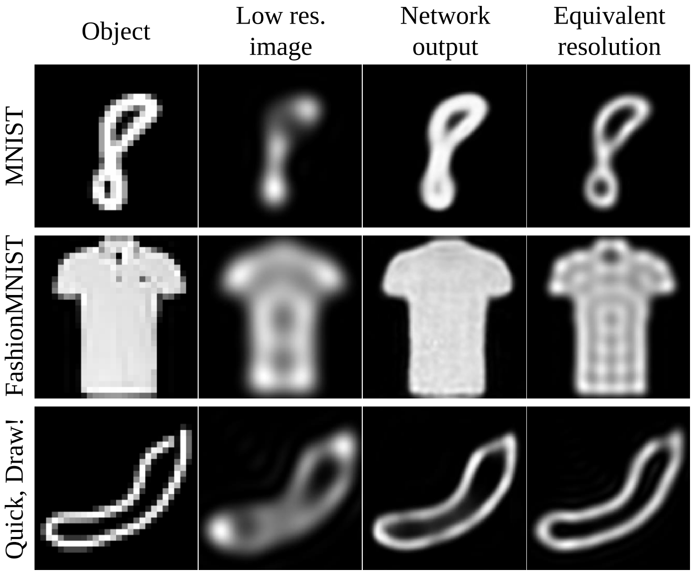

Fig. S4: Qualitative comparison between the output of a trained AOSRNN and the images produced by equivalent imaging optics. Results are shown for the MNIST, FashionMNIST and Quick, Draw! datasets. The outputs of AOSRNN are not always visually similar to the images produced by an equivalent imaging optics, but they achieve the same PSNR.

Supplementary Note 4 AOSRNN performance for input images of different resolution

To evaluate the capabilities of AOSRNN we trained it with input images of different resolutions. The input images were obtained at the output plane of imaging optics with NA of approximately $\{0.01, 0.006, 0.004, 0.003\}$. This NA corresponds to resolution at the input plane of $\Delta x = \{51.7, 84, 134.4, 168\}$ μm. We trained four different networks, each for input images of different resolution, for the MNIST, FashionMNIST and Quick, Draw! datasets. The networks described in this note were trained without the energy preservation regularization term.

Qualitative results for the MNIST and FashionMNIST datasets are shown in Fig. S5a. The results illustrate that AOSRNN can enhance image resolution even when the input resolution is quite low. However, the performance naturally drops as the input resolution decreases. For example, as seen in the rightmost column of Fig. S5a, when the input images are obtained from imaging optics with NA of approximately 0.003, the low-resolution images lose many of their distinctive attributes. Therefore, feeding such low-resolution images as inputs to AOSRNN may result in output images depicting different contents, as in the case of the digit 5. This is not a unique property of AOSRNN, but rather an inherent limitation in recovering lost information from highly-corrupted measurements.

Figure S5b shows the average resolution improvement for the MNIST, FashionMNIST and Quick, Draw! datasets, as a function of the input resolution. The black diagonal dashed line shows the resolution of the low-resolution images. Several example low-resolution images for the MNIST and FashionMNIST datasets are shown in Fig. S5a. The color markers show the resolution achieved by AOSRNN. The average resolution improvement is $\{1.8, 2, 2.6, 3\}$ for input images of resolution $\Delta x = \{51.7, 84, 134.4, 168\}$ μm, respectively. AOSRNN significantly improves the resolution of the input images, for a range of input resolutions and for all datasets.

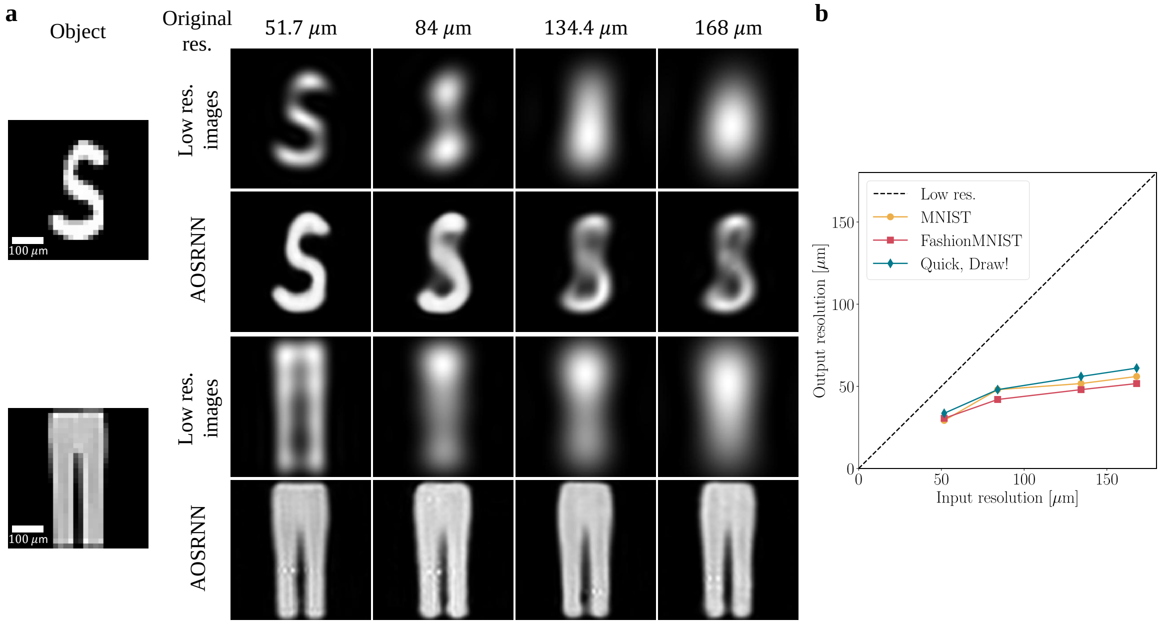

Fig. S5: AOSRNN results for input images of different resolutions. (a) Qualitative examples of outputs of AOSRNN trained on images with different resolution, for the MNIST (top) and FashionMNIST (bottom) datasets. Each column shows the results of a different network. (b) Output resolution vs. input resolution. The black diagonal dashed line shows the resolution of the low-resolution images. The color markers show the resolution at the output of AOSRNN. Each color represents a different dataset, mentioned in the legend.

Supplementary Note 5 Energy loss in nonlinear optical systems

This note provides an explanation for the energy loss inherent to nonlinear optical systems that enhance spatial resolution. Specifically, we take a frequency domain perspective, and demonstrate that in order to accurately recover the lost high frequencies, the new high-frequency components generated by some of the nonlinear layers must be supported on a disk that is larger than the collection cone of the following optical elements. Therefore, energy loss is inevitable.

We analyze an AOSRNN composed of consecutive convolutional units, each containing a $4f$ system with a learned phase mask followed by a nonlinear layer (see Sec. 2). For the simplicity of the analysis, we assume perfect lenses of infinite apertures, so that each $2f$ component of the $4f$ system performs a perfect Fourier transform. We further assume that the nonlinear layers have infinite apertures. In this setting, energy can be lost only at the phase mask planes, which we assume to have a finite circular aperture corresponding to a low-pass filter of radius h in the frequency domain. We will see that even in this favorable scenario, energy loss is unavoidable.

Under the above assumptions, AOSRNN is a shift-invariant system. The $4f$ components perform linear shift-invariant filtering, and thus do not modify the support of the frequency transform of the propagating image. The nonlinear layers are also shift-invariant, but their nonlinearity does cause the frequency support to change. Since energy loss occurs when the frequency range exceeds a phase-mask aperture, and since the phase masks do not change the frequency range, it suffices to analyze spatially-uniform phase masks with all-zero phase. In this case, the phase mask planes affect the propagating image only through their finite aperture.

The input to AOSRNN, $E_0(x_0, y_0)$, which is also the input to the first convolutional unit, is the result of diffraction-limited imaging optics. This imaging optics functions as a low-pass filter in the form of a circ$(\cdot)$ function with radius r (see Supplementary Note 11). Hence, the Fourier transform of this input, denoted by $E_0^{\mathcal{F}}(\nu_{x_0}, \nu_{y_0})$, is supported on a disk of radius r, where we assume that $r < h$.

The first $4f$ system performs perfect imaging in this simplified setting. Therefore, the output of the first convolutional unit is affected only by the nonlinear layer, and is given by

$$E_1(x_1, y_1) = E_0(x_1, y_1) \exp\left\{-j\beta\left(n_0 + n_2|E_0(x_1, y_1)|^2\right)\right\}. \tag{S4}$$

Here we denoted $\beta = \frac{2\pi d}{\lambda}$ where d is the thickness of the nonlinear layer. Using a Taylor expansion of the exponent function in Eq. (S4), this expression can be written as

$$\begin{aligned} E_1(x_1, y_1) &= \exp\{-j\beta n_0\} E_0(x_1, y_1)\left(1 - j\beta n_2|E_0(x_1, y_1)|^2 + \mathcal{O}\left(n_2^2|E_0(x_1, y_1)|^4\right)\right) \\ &\approx \exp\{-j\beta n_0\}\left(E_0(x_1, y_1) - j\beta n_2 E_0(x_1, y_1) E_0(x_1, y_1)\overline{E_0(x_1, y_1)}\right), \end{aligned} \tag{S5}$$

where $\overline{(\cdot)}$ denotes complex conjugation.

The output of the first convolutional unit, E_1, is then used as input to the next convolutional unit, which begins with the Fourier transform of E_1, denote as $E_1^{\mathcal{F}}$,

$$\begin{aligned} E_1^{\mathcal{F}}(\nu_{x_1}, \nu_{y_1}) &= \exp\{-j\beta n_0\}\times \\ &\left(E_0^{\mathcal{F}}(\nu_{x_1}, \nu_{y_1}) - j\beta n_2 E_0^{\mathcal{F}}(\nu_{x_1}, \nu_{y_1}) * E_0^{\mathcal{F}}(\nu_{x_1}, \nu_{y_1}) * \overline{E_0^{\mathcal{F}}(-\nu_{x_1}, -\nu_{y_1})}\right), \end{aligned} \tag{S6}$$

where $*$ denotes the convolution operation. Because of the convolution, the support of $E_1^{\mathcal{F}}$ is three times larger than the support of $E_0^{\mathcal{F}}$ [15],

$$\mathrm{supp}\{E_1^{\mathcal{F}}\} = 3\,\mathrm{supp}\{E_0^{\mathcal{F}}\} = 3r. \tag{S7}$$

The amount of energy lost in this convolutional unit depends on the relation between $3r$ and h. If $3r > h$, then the energy loss is amounted to the energy summed in a ring with outer radius $3r$ and inner radius h. If $3r \leq h$, then there is no energy loss. However, following the same derivation, each of

the subsequent units will increase the radius by another factor of 3. Therefore energy loss will start occurring at the first layer n for which $3^n r > h$.

If the network is taken to be shallow (with number of layers not exceeding $\log_3(\frac{h}{r})$), then no energy will be lost. However, such a network does not increase the frequency content up to its theoretical upper limit of h. Namely, it does not fulfill its theoretical potential for increasing resolution. In practice, the number of layers needs to be significantly larger than $\log_3(\frac{h}{r})$ for achieving good results. This because it is not enough to merely fill up the entire aperture of the phase masks; The frequency content within the aperture has to be shaped correctly to form an accurate approximation of the high-resolution object. This shaping of the frequency content requires multiple passes through optimized phase-masks, interleaved by nonlinear operations. Therefore, the deeper the network, the better its (normalized) output can be made to resemble the ground-truth object. But this inevitably comes at the cost of reduced energy preservation.

This derivation shows that optically improving resolution of an input signal using nonlinear layers will result in energy loss due to the optical system finite size. This result does not specify the amount of energy loss in the process, but indicates that energy loss will inevitably occur.

Supplementary Note 6 Additional results on out-of-distribution data

This note provides additional results for exploring how a trained AOSRNN performs on out-of-distribution data. This setting is discussed in Sec. 4 of the main text.

Figure S6 shows the phase masks of networks trained using the MNIST, FashionMNIST and Quick, Draw! datasets, and the phase masks of a network trained on all three datasets. Although the phase masks share some similarities, each set of phase masks is unique, resulting from the underlying statistics of the used training data.

Figure S7 is an extended version of Fig. 6 from the main text. This figure includes the results on additional out-of-distribution data in the form of two additional datasets – the KMNIST dataset of handwritten Japanese letters [16] and the EMNIST dataset of handwritten English letters [17]. Both datasets were not part of the training set of *any* of the trained networks. The networks trained on only one dataset (either MNIST, FashionMNIST or the Quick, Draw! datasets) were not able to improve the low-resolution images of the KMNIST dataset. The network trained on the Quick, Draw! dataset was not able to improve the low-resolution images of the EMNIST dataset. However, the network trained on all three datasets was able to improve the low-resolution images for both the KMNIST and EMNIST datasets.

For both the KMNIST and EMNIST datasets we used the drop-in replacement for the MNIST dataset, which also contains 10 classes of 28×28 grayscale images. The same preprocessing described in Sec. 5 in the main text was used on the images. Figure S7a also includes the results of the trained networks on a resolution test of line pairs, where the lines are 2 μm wide and are 8 μm apart.

Figure S7b reports the resolutions achieved by the different networks. Figure S7c reports the difference in PSNR between the networks' outputs and the low resolution images. Positive values indicate improvement w.r.t. the low-resolution inputs, whereas negative values indicate degradation w.r.t the low-resolution inputs.

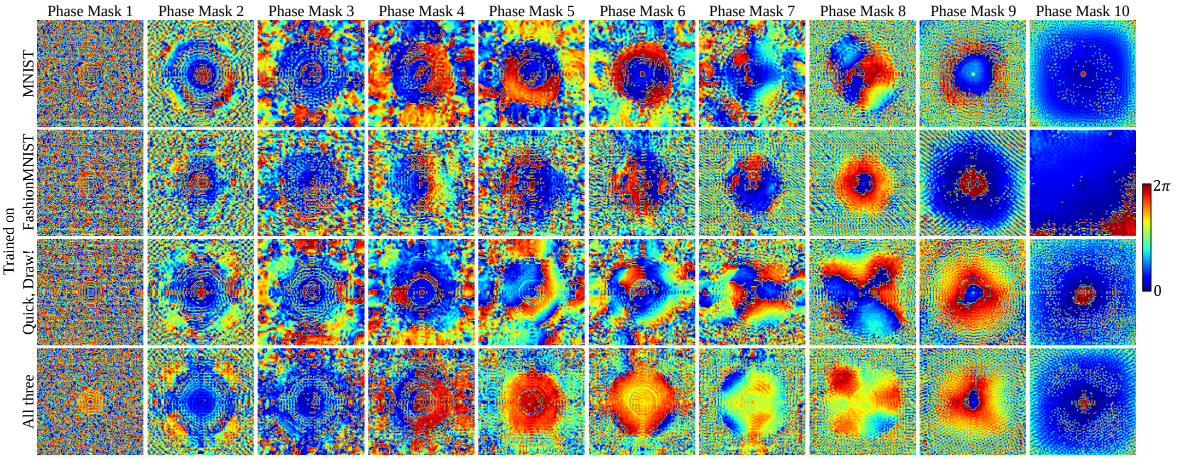

Fig. S6: Learned phase masks of AOSRNNs trained on different datasets. The phase masks are shown with values ranging from 0 to 2π.

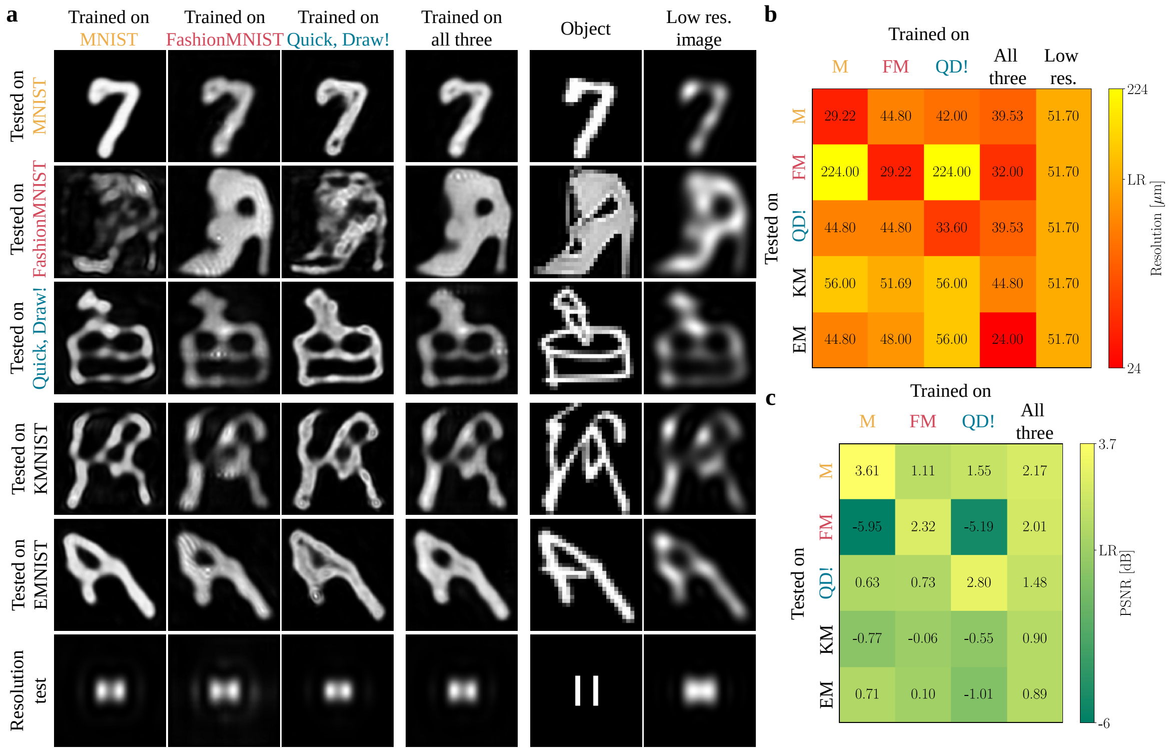

Fig. S7: AOSRNN results on out-of-distribution data. (a) Qualitative results of networks trained on the MNIST, FashionMNIST and Quick, Draw! datasets, as well as a network trained on all these three datasets. These networks were then evaluated on the MNIST, FashionMNIST and Quick, Draw! datasets, as well as on the KMNIST and EMNIST datasets (these datasets were not used as the training data of *any* of the networks) and a resolution test of line pairs. All the results are given next to the high resolution object and the low resolution image. (b) Resolution results of all networks when evaluated on the different datasets. (c) PSNR results of all networks w.r.t the low resolution images, when evaluated on the different datasets.

Supplementary Note 7 The cost of immunity to all sensitivities simultaneously

This note explores exposing AOSRNN to diverse training conditions to achieve immunity to all the sensitivities described in the main text (Secs. 3, 4). We trained a network using all datasets (to reduce sensitivity to out-of-distribution data), with multiple intensity values $p \in [0, 4]$ (to reduce sensitivity to the input intensity), and with the energy preservation regularization term (to reduce energy loss). We refer to this network as "All+IR" (all datasets and intensity robust).

The "All+IR" network performs worse than a network trained with a single objective (*i.e.*, one dataset, specific intensity conditions). Figure S8 shows the PSNR results for different networks relative to the PSNR of the low-resolution images. The first three rows of Fig. S8a, middle panel, illustrate that a specialized network always outperforms the "All+IR" network when evaluated with the specific conditions with which the specialized network was trained. This can be seen by comparing the diagonal values to the values at the rightmost column for these three rows. However, the rest of the cells of Fig. S8a show that this network performs best for different conditions, both for different datasets and for different maximal intensity values.

Figure S8b compares the PSNR results of an IR network trained on the MNIST dataset with those of the "All+IR" network. While the former outperforms the "All+IR" network when evaluated on the MNIST dataset, it performs significantly worse when evaluated on any other dataset.

The "All+IR" network always performs worse when evaluated against a specialized network on the conditions used to train the later. In addition, although the "All+IR" network is robust to intensity changes and generalizes well to different datasets, it still does not outperform the low-resolution measurements for all different conditions. We believe that this can be resolved, at least partially, by making the network deeper (*i.e.*, adding more convolutional units).

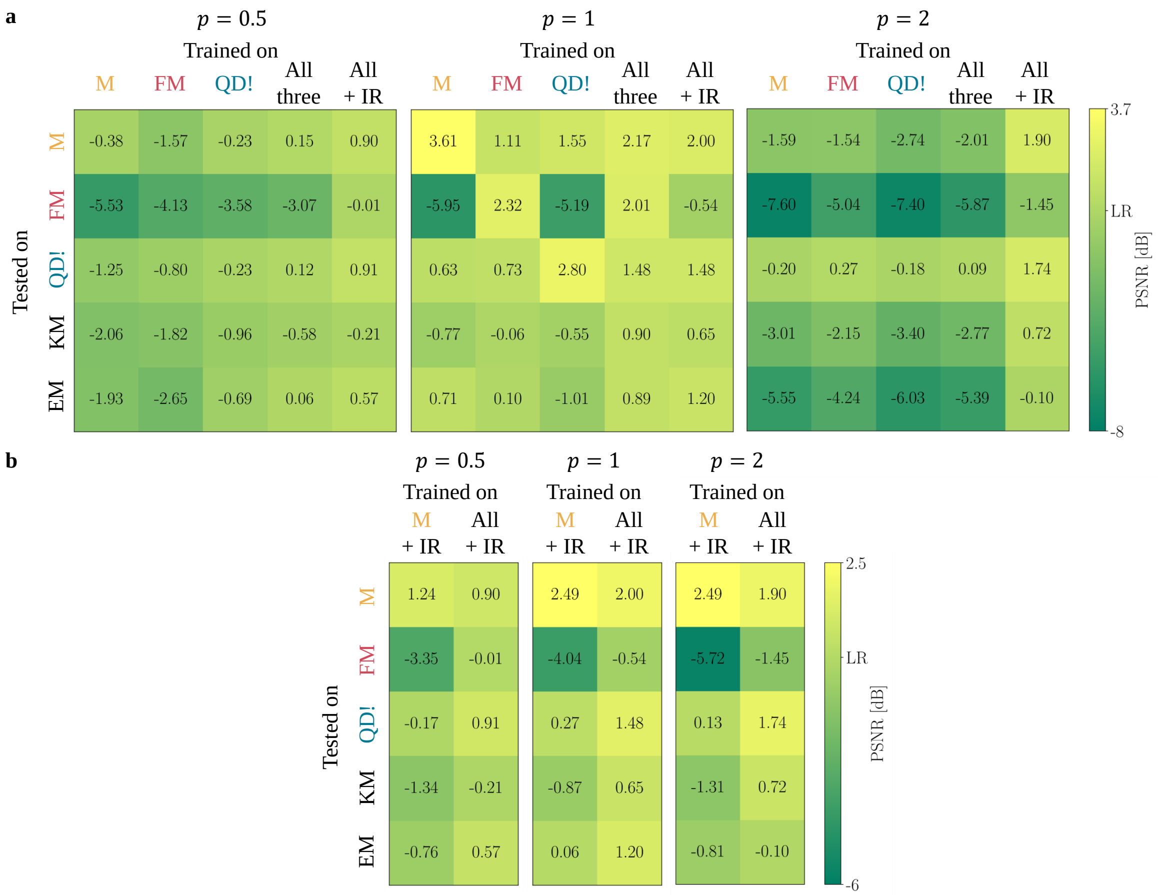

Fig. S8: The cost of immunity to all sensitivities simultaneously. (a) PSNR results of networks trained with $p = 1$, w.r.t the low resolution images, when evaluated on multiple datasets and for $p = \{0.5, 1, 2\}$. The results of a network trained to be immune to all sensitivities ("All+IR") are given in the last column of each pane. (b) Same as (a) but for a network trained to be only intensity-robust (IR) on the MNIST dataset and the "All+IR" network.

Supplementary Note 8 All-optical passive nonlinear neural networks for classification

In the main text we primarily addressed all-optical passive nonlinear neural networks for spatial super-resolution. However, the tradeoffs we explore are inherently linked to the properties of light, and may thus arise in different tasks as well. Here we exemplify it by training a diffractive network with the AOSRNN architecture, but for image classification [14, 18].

The inputs to the network are regular objects that were not first imaged using imaging optics with limited NA. These objects underwent the same preprocessing described in Sec. 5 of the main text. The output plane of the network was divided into predetermined regions, each corresponding to a different class. The region with the highest intensity read was chosen as the predicted class [19].

When trained on the MNIST dataset, this network achieves 94.8% test accuracy. However, this is achieved by scattering a significant portion of the incoming light. The network used its nonlinear layers to learn a complex nonlinear mapping between input and output. This learned complex nonlinear mapping is implemented by generating high-spatial frequencies that inevitably escape the network's limited NA. As suggested in the main text, adding a regularization term reveals a tradeoff between classification accuracy and energy preservation, as illustrated in Fig. S9a. For example, the regularization term can be used to obtain a network that preserves $\sim 40\%$ of the total energy. But this network achieves only 91.1% classification accuracy.

When evaluating this network with maximal intensity values (again, denoted by p) different than the one used for training ($p = 1$), its performance quickly deteriorates. For example, it exhibits a drop of 33% in classification accuracy when increasing the intensity by a factor of 2. As explained in the main text, exposing a network to images with different p values during training results in intensity-robust (IR) network. Although the IR network (trained with $p \in [0, 8]$) achieves lower maximal classification accuracy (81.6%, which is $\sim 9\%$ drop in accuracy w.r.t the accuracy achieved by a network trained and evaluated with $p = 1$) it achieves relatively consistent performance for various p values, as illustrated in Fig. S9b.

These results illustrate that while incorporating an all-optical nonlinear layer into a passive diffractive network trained for different machine learning tasks is desirable [1, 2], it also introduces fundamental tradeoffs that must be carefully balanced in the network's design.

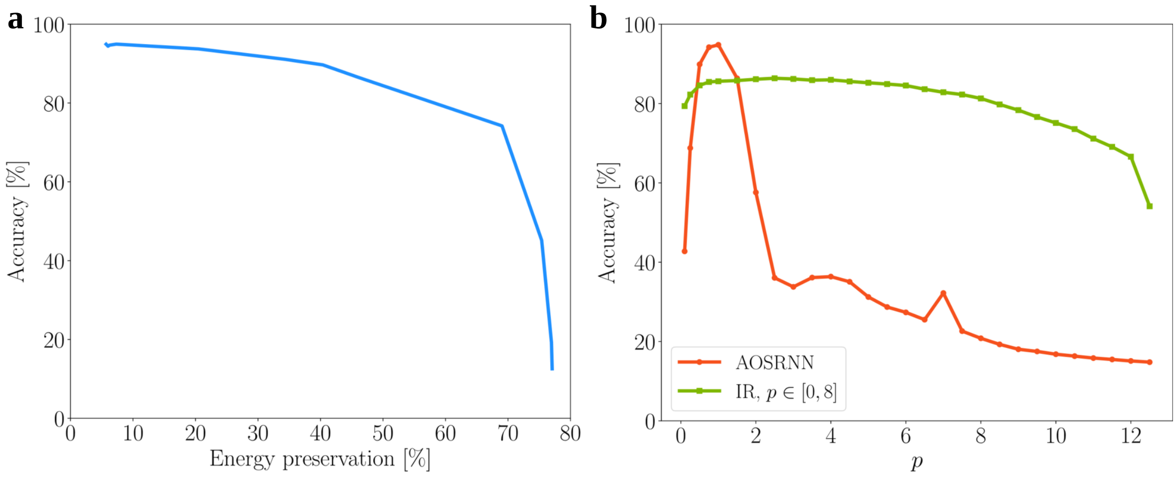

Fig. S9: Tradeoffs of all-optical passive nonlinear network for classification. (a) Energy preservation vs. classification accuracy. (b) Sensitivity to global intensity. The results of a network trained with $p = 1$ in orange, and the results of intensity-robust (IR) network, in green.

Supplementary Note 9 A different diffractive neural network architecture

To illustrate that the identified tradeoffs (energy preservation vs. resolution and sensitivity to global intensity) are not the result of a specific architecture, we present here the results of a nonlinear passive deep diffractive neural network [18] designed for all-optical spatial super-resolution. This network architecture involves free space propagation between consecutive diffractive layers, where each diffractive layer modulates the optical field. To adjust this architecture to all-optical spatial super-resolution, we added between each consecutive phase masks a nonlinear layer, separated by free space propagation (Fig. S10a).

We trained a 10 layer network with this architecture on the MNIST dataset. The trained network successfully enhances the input resolution, achieving a PSNR of 17.4 dB for input images with PSNR of 16.1 dB (see qualitative results in Fig. S10b). However, similarly to the AOSRNN discussed in the main text, this network suffers from the same identified tradeoffs. Specifically, only $\sim 1\%$ of the input energy is preserved in the output plane. Furthermore, when multiplying the input intensity by 2 or 4 the PSNR deteriorates to 16.6 dB or 14.2 dB, respectively.

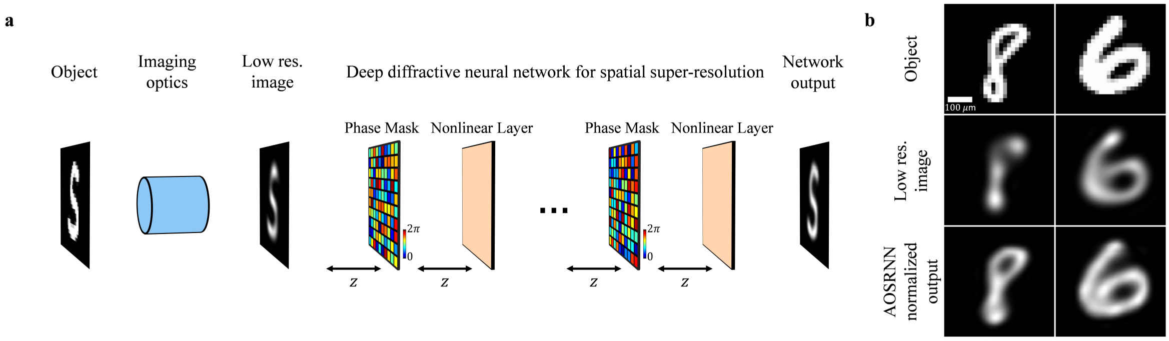

Fig. S10: A different nonlinear passive diffractive neural network architecture for all-optical super resolution. (a) Network architecture. (b) Qualitative results on the MNSIT dataset.

The propagation through this network involves free space propagation, diffractive and nonlinear layers. The free space propagation was simulated using the Rayleigh-Sommerfeld diffraction formulation and the angular spectrum method [15]. All fields were adequately padded to prevent aliasing. The Rayleigh-Sommerfeld transfer function is

$$H_{\text{R-S}}(f_x, f_y; z, \lambda) = \begin{cases} \exp\left\{ j\frac{2\pi}{\lambda} z \sqrt{1-\lambda^2(f_x^2+f_y^2)} \right\} & \text{if } \sqrt{f_x^2+f_y^2} \leq \frac{1}{\lambda}, \\ 0 & \text{otherwise,} \end{cases} \tag{S8}$$

where λ is the wavelength, z is the propagation distance, f_x and f_y are the spatial frequencies along the x and y directions, respectively. Using this transfer function, we can write the electromagnetic field after propagation by a distance d, as

$$E(x, y; z+d, \lambda) = \mathcal{F}^{-1}\{\mathcal{F}\{E(x', y'; z, \lambda)\} \cdot H_{\text{R-S}}(f_x, f_y; d, \lambda)\}, \tag{S9}$$

where $\mathcal{F}$ and $\mathcal{F}^{-1}$ are the two-dimensional Fourier transform and inverse Fourier transform operations, respectively. We used $\lambda = 550$ nm and $z = 1$ cm.

The phase masks and nonlinear layers are implemented in the same way as in our AOSRNN, and have the same dimensions (see Supplementary Note 11 for details).

Supplementary Note 10 Polychromatic illumination

This note illustrates the results of an AOSRNN for the MNIST dataset illuminated by polychromatic light comprising three different wavelengths – $\{400, 550, 700\}$ nm. Training with polychromatic light instead of monochromatic light leads to two differences, resulting from the different wavelengths. The first difference is in the input resolution. The radius of the ideal $\mathrm{circ}(\cdot)$ transfer function of the limited-NA imaging system is inversely dependent on the illumination wavelength (Supplementary Note 11). Therefore, for polychromatic illumination, each wavelength corresponds to a different input resolution. Specifically, for the simulated wavelengths, $\lambda = \{400, 550, 700\}$ nm, the input resolution is $\Delta x = \{37.6, 51.7, 65.8\}$ μm, respectively, resulting, in input images with PSNR of 16.6 dB, 0.5 dB higher than the input resolution when illuminated by monochromatic illumination of $\lambda = 550$ nm. The second difference occurs in the nonlinear layers. In these layers, the phase depends on the intensity, which is the sum of intensities in the different wavelengths, $I(x, y) = \sum_\lambda |E(x, y; \lambda)|^2$. This results in some cross-influence between the wavelengths.

This network achieves PSNR of 20.65 dB, which is 0.8 dB higher than the resolution achieved by AOSRNN trained with monochromatic light of $\lambda = 550$ nm. See Fig. S11 for qualitative results. However, it still suffers from the identified tradeoffs. Specifically, only $\sim 1\%$ of the input energy is preserved in the output plane. Additionally, when multiplying the input intensity by 2 or 4, the PSNR deteriorates to 14.3 dB and 11.1 dB, respectively.

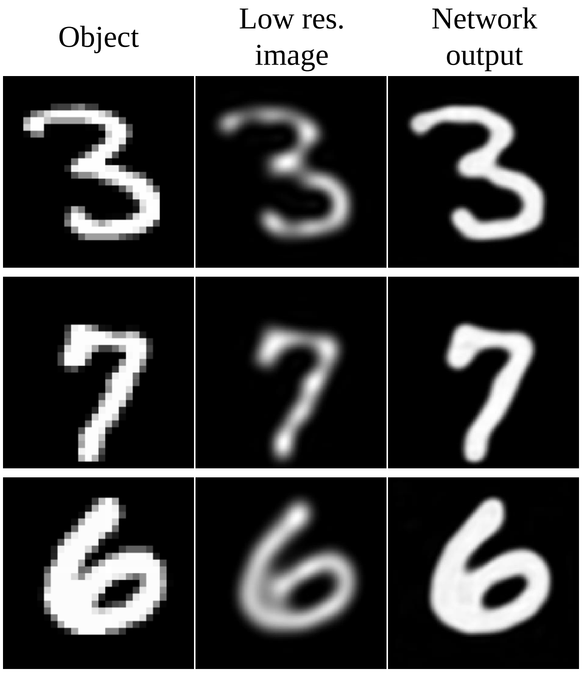

Fig. S11: AOSRNN trained with polychromatic illumination. Qualitative results on the MNSIT dataset.

Supplementary Note 11 Forward propagation through AOSRNN

The input to the AOSRNN is low-resolution images obtained at the output plane of a limited NA imaging optics. We simulate these low-resolution images by using a low-pass filter in the frequency domain, following

$$E_{\mathrm{LR}}^{\mathcal{F}}(\nu_x,\nu_y)=H(\nu_x,\nu_y)\cdot E_{\mathrm{HR}}^{\mathcal{F}}(\nu_x,\nu_y), \tag{S10}$$

where $E_{\mathrm{HR}}^{\mathcal{F}}$ is the Fourier transform of the object, the input to imaging optics, and $E_{\mathrm{LR}}^{\mathcal{F}}$ is the Fourier transform of the low resolution image in its output plane. The low pass filter in the frequency domain is the amplitude transfer function, denoted by H, of a coherent, diffraction limited, system [15]. In the numerical experiments we simulated imaging optics with circular aperture, whose amplitude transfer function is [15]

$$H(\nu_x,\nu_y)=\mathrm{circ}\left(\frac{\sqrt{\nu_x^2+\nu_y^2}}{r_p/\lambda z_i}\right), \tag{S11}$$

where ν_x and ν_y are the frequency domain coordinates, r_p is the radius of a circular pupil in the space domain, λ is the used wavelength and z_i is the distance between the exit pupil to the output plane. The circ function is defined as

$$\mathrm{circ}(r)=\begin{cases}1 & r<1,\\ 0 & \text{otherwise.}\end{cases} \tag{S12}$$

For ν_x we used a uniform grid over $[-\frac{1}{2p_x},\frac{1}{2p_x}]$, where the step size is given as $\frac{1}{O_x\cdot p_x}$. Here, p_x is the width dimension of each pixel, and O_x is the number of pixels along the width dimension of the object. Similarly, ν_y is identically defined along the height dimension. As we used symmetric coordinates systems, the grids of ν_x and ν_y are the same.

All the results in the main text are given with $\lambda = 550$ nm, $z_i = 1$ cm, $p_x = p_y = 4$ μm and $O_x = O_y = 112$. Input resolutions of $\{51.7, 84, 134.4, 168\}$ μm correspond to $r_p = \{60, 100, 140, 180\}^{-1}$ cm.

The low-resolution images are then used as input to the AOSRNN. This network is comprised of consecutive convolutional units, comprised of $4f$ systems followed by all-optical nonlinear layers.

The all-optical convolution layers are achieved by positioning a learnable phase mask in the Fourier plane,

$$E_{\mathrm{out}}(x',y')=\mathcal{F}^{-1}\{\mathcal{F}\{E_{\mathrm{in}}(x,y)\}(\nu_x,\nu_y)\cdot e^{-j\phi(\nu_x,\nu_y)}\}(x',y'), \tag{S13}$$

where $\mathcal{F},\mathcal{F}^{-1}$ are the 2-dimensional Fourier transform and inverse Fourier transform, respectively, E_{in} is the input field which its Fourier transform is being multiplied by a learnable phase mask, ϕ, and E_{out} is the output field. All fields were adequately zero padded to avoid aliasing effect.

We used a nonlinear refractive index as the nonlinear mechanism in the numerical experiments, unless specifically stated otherwise. It is further discussed in Sec. 2 in the main text and in Supplementary Note 1.

At the output of the AOSRNN we calculate the intensity of the output field, recorded by a sensor as

$$I(x,y)=|E(x,y)|^2. \tag{S14}$$

References

[1] G. Wetzstein, A. Ozcan, S. Gigan, S. Fan, D. Englund, M. Soljačić, C. Denz, D. A. Miller, and D. Psaltis, "Inference in artificial intelligence with deep optics and photonics," *Nature*, vol. 588, no. 7836, pp. 39–47, 2020.

[2] P. L. McMahon, "The physics of optical computing," *Nature Reviews Physics*, vol. 5, no. 12, pp. 717–734, 2023.

[3] D. Zhang, D. Xu, Y. Li, Y. Luo, J. Hu, J. Zhou, Y. Zhang, B. Zhou, P. Wang, X. Li, *et al.*, "Broadband nonlinear modulation of incoherent light using a transparent optoelectronic neuron array," *Nature Communications*, vol. 15, no. 1, p. 2433, 2024.

[4] D. N. Christodoulides, I. C. Khoo, G. J. Salamo, G. I. Stegeman, and E. W. Van Stryland, "Nonlinear refraction and absorption: mechanisms and magnitudes," *Advances in Optics and Photonics*, vol. 2, no. 1, pp. 60–200, 2010.

[5] I.-C. Khoo, *Nonlinear Optical Processes Observed in Liquid Crystals*, ch. 12, pp. 348–397. John Wiley & Sons, Ltd, 2022.

[6] A. A. Majd, C. Amaljith, and A. Ibrahim, "Enhanced speed and tunability of liquid crystals in nanocavities via engineering the local electromagnetic field," *ACS Photonics*, vol. 12, no. 2, pp. 908–916, 2025.

[7] N. Kinsey, C. DeVault, J. Kim, M. Ferrera, V. Shalaev, and A. Boltasseva, "Epsilon-near-zero al-doped zno for ultrafast switching at telecom wavelengths," *Optica*, vol. 2, no. 7, pp. 616–622, 2015.

[8] M. Z. Alam, I. De Leon, and R. W. Boyd, "Large optical nonlinearity of indium tin oxide in its epsilon-near-zero region," *Science*, vol. 352, no. 6287, pp. 795–797, 2016.

[9] P. Vabishchevich and Y. Kivshar, "Nonlinear photonics with metasurfaces," *Photonics Research*, vol. 11, no. 2, pp. B50–B64, 2023.

[10] C. U. Hail, L. Michaeli, and H. A. Atwater, "Third harmonic generation enhancement and wavefront control using a local high-q metasurface," *Nano Letters*, vol. 24, no. 7, pp. 2257–2263, 2024.

[11] D. Milam, M. J. Weber, and A. Glass, "Nonlinear refractive index of fluoride crystals," *Applied Physics Letters*, vol. 31, no. 12, pp. 822–825, 1977.

[12] R. Adair, L. Chase, and S. A. Payne, "Nonlinear refractive index of optical crystals," *Physical Review B*, vol. 39, no. 5, p. 3337, 1989.

[13] Q. Feng, C. B. Uzundal, R. Guo, C. Sanborn, R. Qi, J. Xie, J. Zhang, J. Wu, and F. Wang, "Femtojoule optical nonlinearity for deep learning with incoherent illumination," *Science Advances*, vol. 11, no. 5, p. eads4224, 2025.

[14] M. Kleiner, L. Michaeli, and T. Michaeli, "Coherence awareness in diffractive neural networks," *Laser & Photonics Reviews*, vol. 19, no. 10, p. 2401299, 2025.

[15] J. W. Goodman, *Introduction to Fourier optics*. Roberts and Company publishers, 2005.

[16] T. Clanuwat, M. Bober-Irizar, A. Kitamoto, A. Lamb, K. Yamamoto, and D. Ha, "Deep learning for classical japanese literature," *Machine Learning for Creativity and Design Workshop in Advances in Neural Information Processing Systems*, 2018.

[17] G. Cohen, S. Afshar, J. Tapson, and A. Van Schaik, "Emnist: Extending mnist to handwritten letters," in *2017 international joint conference on neural networks (IJCNN)*, pp. 2921–2926, IEEE, 2017.

[18] X. Lin, Y. Rivenson, N. T. Yardimci, M. Veli, Y. Luo, M. Jarrahi, and A. Ozcan, "All-optical machine learning using diffractive deep neural networks," *Science*, vol. 361, no. 6406, pp. 1004–1008, 2018.

[19] M. Kleiner, L. Michaeli, and T. Michaeli, "Coherence awareness in diffractive neural networks," in *CLEO 2024*, p. FW4Q.5, Optica Publishing Group, 2024.
